# Highly Stable Thin Films Based on Novel Hybrid 1D (PRSH)PbX_3_ Pseudo-Perovskites

**DOI:** 10.3390/nano11102765

**Published:** 2021-10-19

**Authors:** Gabriele Calabrese, Candida Pipitone, Diego Marini, Francesco Giannici, Antonino Martorana, Luisa Barba, Caterina Summonte, Norberto Masciocchi, Silvia Milita

**Affiliations:** 1 Istituto per la Microelettronica e Microsistemi, Consiglio Nazionale delle Ricerche, via Gobetti 101, 40129 Bologna, Italy; marini@bo.imm.cnr.it (D.M.); summonte@bo.imm.cnr.it (C.S.); 2 Dipartimento di Fisica e Chimica, Università di Palermo, viale delle Scienze, Ed. 17, 90128 Palermo, Italy; candidapipitone@gmail.com (C.P.); francesco.giannici@unipa.it (F.G.); antonino.martorana@unipa.it (A.M.); 3 Istituto di Cristallografia, Consiglio Nazionale delle Ricerche, Strada Statale 14-km 163, 5, AREA Science Park, Basovizza, 34149 Trieste, Italy; luisa.barba@ic.cnr.it; 4 Dipartimento di Scienza e Alta Tecnologia e To.Sca.Lab., Università dell’Insubria, via Valleggio 11, 22100 Como, Italy

**Keywords:** hybrid-halide perovskites, 1D perovskites, GIXD, SEM imaging, thin films, humidity resistant

## Abstract

In this study, the structure and morphology, as well as time, ultraviolet radiation, and humidity stability of thin films based on newly developed 1D (PRSH)PbX_3_ (X = Br, I) pseudo-perovskite materials, containing 1D chains of face-sharing haloplumbate octahedra, are investigated. All films are strongly crystalline already at room temperature, and annealing does not promote further crystallization or film reorganization. The film microstructure is found to be strongly influenced by the anion type and, to a lesser extent, by the DMF/DMSO solvent volume ratio used during film deposition by spin-coating. Comparison of specular X-ray diffraction and complementary grazing incidence X-ray diffraction analysis indicates that the use of DMF/DMSO mixed solvents promotes the strengthening of a dominant 100 or 210 texturing, as compared the case of pure DMF, and that the haloplumbate chains always lie in a plane parallel to the substrate. Under specific DMF/DMSO solvent volume ratios, the prepared films are found to be highly stable in time (up to seven months under fluxing N_2_ and in the dark) and to highly moist conditions (up to 25 days at 78% relative humidity). Furthermore, for representative (PRSH)PbX_3_ films, resistance against ultraviolet exposure (λ = 380 nm) is investigated, showing complete stability after irradiation for up to 15 h at a power density of 600 mW/cm^2^. These results make such thin films interesting for highly stable perovskite-based (opto)electronic devices.

## 1. Introduction

Perovskites have recently emerged as the holy grail of photovoltaics, reaching in a decade of research a solar cell certified efficiency of 25.5% for a single-junction device, thus rivaling the performance of well-established technologies [1]. Despite their excellent performances [2,3,4], which have been exploited in a large number of (opto)electronic devices well beyond solar cells (such as gas sensors [5], transistors [6], thermoelectric [7] and piezoelectric [8] generators, light-emitting diodes [9], lasers [10], and photodetectors [11]), the reduced long-term stability of the large majority of inorganic and hybrid perovskites still represents the main obstacle preventing their commercialization and, therefore, their optimization is one of the main research topics in the field [12,13]. The limited stability of 3D perovskites has been attributed to the low material formation energy, which results in the strong propensity of the formed crystals to decompose back into their precursors [14], particularly if the organic cation decomposition generates gaseous species [such as methylamine from methylammonium lead iodide, (CH_3_NH_3_)PbI_3_ or MAPI]. To enhance material stability, low-dimensional 2D perovskite counterparts have been investigated, showing much better stability under ambient conditions [15,16], a result that was ascribed to their increased formation energy [17]. Much work has been done by the research community on 2D perovskites, and it has become clear that a trade-off between (opto)electronic performances and time stability exists, the former being maximized in 3D materials with the latter being favored in 2D counterparts [17]. In the search for an optimized strategy, devices based on hybrid 2D/3D composites have been proposed (showing impressive results) [18,19]. Despite these achievements, it is still not clear whether 2D perovskites have the potential to provide ultimate material stability thus enabling commercialization of perovskite-based devices. Indeed, such improved stability in time and towards environmental moisture is shared by all perovskite-based materials showing reduced dimensionality (2D, 1D, 0D) [20,21,22,23,24,25]. In the search for stable materials of this class, it is therefore important to scrutinize for all possible promising alternatives, including 1D structures.

Recently, we reported the isolation of new 1D (C_14_H_19_N_2_)PbX_3_ (X = Br, I) pseudo-perovskite materials prepared by a simple one-pot method [26], where the organic cation, the protonated proton sponge [1,8-Bis(dimethylamino)naphthalene, or PRS], was chosen for its nearly null tendency toward H^+^ release, a typical decomposition path of ammonium and iminium cations [27,28,29]. Another peculiar feature of the compound bis(dimethylamino)naphthalene is the slowness of proton addition—detachment due to the shielding of inter nitrogen space by four methyl groups. Thus, the relatively high thermodynamic basicity of 1,8-bis(dimethylamino)naphthalene is associated with its rather low kinetic basicity [30].

In this work we present the structural and morphological characterization of thin films based on these new compounds, and we report on their excellent stability. Since the effect of the used solvent on the structure, morphology, and final performance of 3D perovskites has been reported to be very important [31,32,33], in this work we investigate the effect of the choice of the solvent (DMF or a mixture of DMF and DMSO at different volume ratios) on the structure and morphology of the newly developed pseudo-perovskites. The results obtained in the present work for the 1D (C_14_H_19_N_2_)PbX_3_ systems and the retrieved general rules can be extended to a wider class of low-dimensional perovskites.

To obtain a complete picture of the investigated systems, we combined specular laboratory X-ray diffraction (XRD) with synchrotron grazing incidence X-ray diffraction (GIXD) measurements. XRD techniques optimized for the structural characterization of thin films are of paramount importance as thin films are required for device fabrication and their (micro)structural properties may differ from those of the bulk materials [34,35]. In-situ GIXD measurements were carried out during annealing to study the rapid structural evolution of a 50/100 nm-thick film, and such analysis required the use of synchrotron radiation in order to be able to observe also weak (reversible) transformation.

The thin films here investigated were prepared on glass substrates by spin-coating and showed high crystallinity without the need of post-deposition thermal treatments, being hence suitable for the direct printing on low softening point bendable substrates. Film texturing and morphology strongly depend on the choice of the halide and, to a lower extent, also to the film preparation conditions. In particular, the effect of the DMF/DMSO solvent volume ratio and the choice of the halide anion were investigated here, as these are known factors in addressing the morphology and structure of 3D perovskites. Finally, aging experiments, photodegradation experiments under ultraviolet (UV) light and exposition to controlled humidity environments, revealed that under specific fabrication conditions the synthesized films are highly stable and might then represent promising materials for future devices.

This work, inter alia, highlights the powerful combination of microscopy imaging and XRD/GIXD studies for the in-depth morphological and structural characterization of low dimensional pseudo-perovskite thin films, which features strongly depend on material composition, preparation conditions, thermal treatment, and exposition to environmental moisture.

The paper is organized as follows. After reporting the description of the used materials and methods in Section 2, we discuss the effect of the employed solvent on both the morphology (Section 3.1) and crystalline structure (Section 3.2) of the fabricated (PRSH)PbX_3_ films as a function of the halide anion, and we show how the 1D chains of face-sharing haloplumbate octahedra arrange at substrate surface, an information retrieved from both XRD and GIXD analysis. In Section 3.3 we discuss the effect of both ex-situ and in-situ thermal annealing on the morphology and structure of the studied films. Finally, we report the film structural evolution in time (Section 3.4), after exposure to UV light (Section 3.5) and to controlled humidity environment (Section 3.6).

## 2. Materials and Methods

### 2.1. Film Preparation

Powders of (PRSH)PbBr_3_ and (PRSH)PbI_3_, prepared as described in Ref. [26] were dissolved at a concentration of 40 mg/mL in DMF and DMF/DMSO mixtures with volume ratios of 4:1, 1:1 and 1:4, by gently stirring the solutions for 3 h at 30 °C. Successively, 50 µL of each solution were spin-coated for 20 s at 4000 rpm using an Ossila (Sheffield, UK) system on 1 mm-thick and 0.5 × 1 cm^2^ wide glass substrates. Before film deposition, the substrates were sequentially sonicated in DI water, acetone and isopropyl alcohol for 10 min each, and finally treated in an ozone cleaner for 20 min.

### 2.2. Annealing, Aging and Exposition to Controlled Humidity Environment

For the annealing experiments, the samples were heated on a hot plate in air at 100 °C for 15 min. To study the time stability of the fabricated films, these were stored for seven months in a desiccator streamed by a weak N_2_ flux and kept in the dark. The stability of the samples toward humidity was investigated by storing the samples at 20 °C in a sealed chamber having a controlled RH% value determined by the presence of saturated salt solutions of K_2_CO_3_ and NaCl (with nominal RH% values of 44 and 78%, respectively).

### 2.3. Photodegradation Experiments

Photodegradation experiments were carried out by exposing representative (PRSH)PbX_3_ films to UV light using a light emitting diode (LED) with emission peak at 380 nm. To investigate the structural stability of the films, we collected consecutive specular X-ray diffraction (XRD, Bruker D8, MA, USA) profiles in-situ, i.e., during sample exposition to a power density of 600 mW/cm^2^ (6 Suns) for a total irradiation time of 15 h, each measurement taking approximately 30 min (λ = 1.5406 Å).

### 2.4. Scanning Electron Microscopy

The morphology of the as-prepared and annealed films was investigated by Scanning electron microscopy (SEM, ZEISS Leo 1530, Oberkochen, Germany) in top view and cross-section configurations, using 5kV accelerating voltage and an in-lens detector. The analysis of the recorded micrographs was carried out by using the open software ImageJ [36].

### 2.5. Grazing Incidence X–ray Diffraction (GIXD) and Specular XRD

To obtain a complete view of the film structure, texturing, mosaicity, and homogeneity across depth, complementary synchrotron GIXD and specular laboratory XRD (Rigaku Smartlab, Tokyo, Japan) techniques were used. Indeed, by combining these two techniques, the different components of the scattering vector q are investigated: in the specular geometry, only the q_z_ contributions (at q_xy_ = 0) are recorded (stemming from lattice planes lying parallel to the substrate surface, only partially detectable by GIXD, as their theoretical positions lie in the blind region of the map). Vice versa, in GIXD only scattering vectors with non-null q_xy_ components can be measured, corresponding to lattice planes tilted with respect to the substrate surface.

GIXD measurements were performed at the XRD1 beamline of the ELETTRA synchrotron radiation facility in Trieste, Italy. 2D-GIXD images were collected by using a 2M Pilatus silicon pixel X-ray detector (DECTRIS Ltd., Baden, Switzerland) positioned perpendicular to the incident beam, 350 mm away from the sample. The wavelength was fixed to λ = 1.00 Å, and a beam size of 200 × 200 μm^2^ was selected. Two different X-ray beam incident angles were chosen: (i) α_i_  =  0.05° to probe the uppermost film layers (the X-ray penetration depth is ~ 4 nm for both film compositions) and (ii) α_i_  =  0.2° to probe the full film thickness (the X-ray penetration depths are ~ 520 and 970 nm for (PRSH)PbI_3_ and (PRSH)PbBr_3_ films, respectively, which are by far larger than the film thickness of ~ 50–100 nm as determined from cross-sectional scanning electron micrographs reported in the Supplementary Materials (Figures S1e–h and S2e–h). In-situ and real time GIXD measurements during sample heating at 130 °C with a hot air flux were performed at α_i_  =  0.2° by collecting one 2D image every 90 sec. The visualization and analysis of all the recorded 2D-GIXD images was performed using the open software GIDVis [37].

The Rigaku Smartlab system was operated in specular (i.e., θ/2θ) geometry using a Cu rotating anode. A graded multilayer mirror was used to produce a parallel X-ray beam (λ = 1.5406 Å) with a divergence ≤0.04° in the diffraction plane, and 5° Soller slits were placed in front of the detector to reduce the angular divergence of the diffracted beam.

### 2.6. Optical Measurements

Reflectance (R) and transmission (T) spectra in the UV-Vis range were acquired using an Avantes (Apeldoorn, Netherlands) fiber optics spectrophotometer equipped with an integrating sphere. From R(%) and T(%), the absorption spectra A(%) = 1 − R(%) − T(%) were calculated. Measurements on bare glass substrates were also taken.

## 3. Results and Discussion

### 3.1. Morphology of the As-Deposited (PRSH)PbX_3_ Thin Films

**Figure 1:**
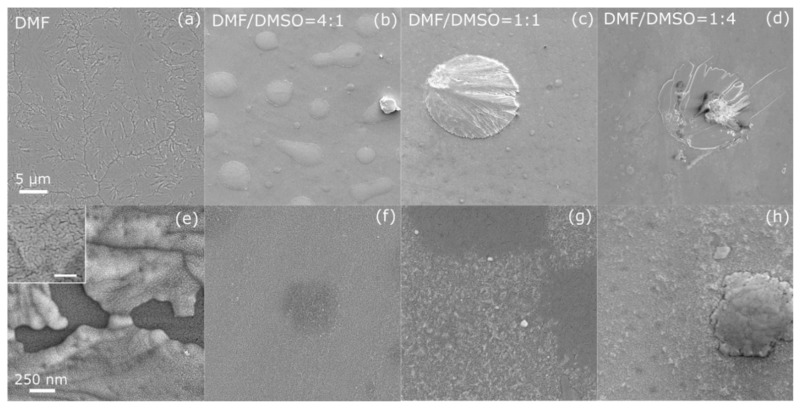
Scanning electron micrographs taken at low (**a**–**d**) and high (**e**–**h**) magnification on (PRSH)PbBr_3_ films prepared in DMF (**a**,**e**) and in mixed DMF/DMSO solvents with a volume ratio of 4:1 (**b**,**f**), 1:1 (**c**,**g**) and 1:4 (**d**,**h**). The scale bars in the left panels apply to all micrographs in the same line. The inset in (**e**) shows a high magnification micrograph of the uppermost portion of the film in (**a**), with a 100 nm scale bar.

**Figure 2:**
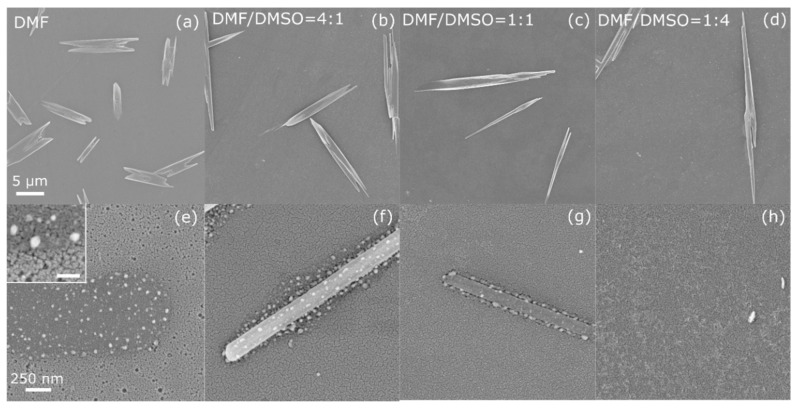
Scanning electron micrographs taken at low (**a**–**d**) and high (**e**–**h**) magnification on (PRSH)PbI_3_ films prepared in DMF (**a**,**e**) and in mixed DMF/DMSO solvents with a volume ratio of 4:1 (**b**,**f**), 1:1 (**c**,**g**) and 1:4 (**d**,**h**). The scale bars in the left panels apply to all micrographs in the same line. The inset in (**e**) shows a high magnification micrograph of the film in (**a**), with a 100 nm scale bar.

The top-view SEM images of the (PRSH)PbBr_3_ and (PRSH)PbI_3_ films (Figure 1 and Figure 2, respectively), taken at low and high magnification, allow us to establish the morphology of the as-prepared films at different length scales, and their dependence on the solvent composition.

The (PRSH)PbBr_3_ film prepared by using DMF as the only solvent exhibits disconnected regions (Figure 1a,e) due to the presence of cracks all over the surface, which are suppressed by adding DMSO to DMF [Figure 1b–d,f–h]. All films consist of small grains, few tens of nm wide, which slightly increase in size when DMSO is added (see the higher magnification SEM images reported Figure S1, Supplementary Materials). Both the disappearance of the cracks and the widening of the grains are attributed to the slower crystallization rate of the films in the presence of DMSO, which possesses a lower vapor pressure than DMF (0.42 and 2.7 Torr at 20 °C, respectively [38]).

However, the use of the binary solvent (DMF/DMSO) induces the formation of several islands (Figure 1b–d) and crystallites (Figure 1g–h) at the film surface, higher in number and size for large(r) DMSO content.

By replacing the bromide ions with the iodide ones (Figure 2), a similar morphology of the underlying film and dependence on solvent composition are observed. The strongest difference between the (PRSH)PbBr_3_ and (PRSH)PbI_3_ films is the presence, on top of the continuous layer, of elongated µm-sized crystallites in the latter in place of islands, whose size and shape depend on the DMF/DMSO ratio (Figure 2a–d). In particular, the average length of the microstructures increases by about one order of magnitude from ~11 to ~120 μm, with the aspect ratio going from ~3.7 to ~80, when moving from pure DMF to a 1:4 DMF/DMSO volume ratio. Also in this case, the film formed with pure DMF solvent is not continuous, due to the presence of tiny holes (Figure 2e and Figure S2a), which are suppressed by adding DMSO, giving rise to smoother film surfaces (Figure 2f–h). The (PRSH)PbI_3_ films consist of grains of irregular shape (shown in the inset of Figure 2e and in Figure S2, Supplementary Materials), similar to those observed for the (PRSH)PbBr_3_ films, but larger in size (see Figures S1 and S2, Supplementary Materials), which widen when DMSO is added.

Despite the different morphological features between Br and I-based films, two common effects emerge upon using a binary DMF/DMSO solvent mixture. Firstly, more homogeneous and continuous films form on the substrate surface, being the formation of cracks or pinholes highly suppressed. Secondly, larger islands or microcrystals form on top of the film surface. These results can be explained by comparing the Lamer diagrams for more (DMF) and less (DMSO) volatile solvents, showing a preferred nucleation of new sites in the former case and a preferred enlargement of already nucleated sites for the latter [39,40]. Moreover, the addition of DMSO in DMF is known to alter the perovskite crystallization process retarding the reaction of the organic cations with PbI_2_ due to the strong complexing force between PbI_2_ and DMSO which induces the formation of PbI_2_—DMSO complexes [41,42]. These complexes in turn promotes the formation of colloids with a narrow size distribution which were reported to promote the formation of films with improved crystallinity, as experimentally observed here [43].

The evolution of the morphology with the DMF/DMSO solvent ratio, observed for both (PRSH)PbBr_3_ and (PRSH)PbI_3_ films, demonstrates that, as already reported for 3D and 2D perovskite thin film counterparts [15,16,44,45], the composition of the starting solution plays a crucial role during film crystallization. However, the nature of the halide ion also influences the film morphology, even when the same solvent used. In fact, the coordination of DMSO to Pb ions leads to the formation of soluble, or even colloidal, metal-halide complexes [43], as the O-Pb interaction can compete with the Pb-I or Pb-Br ones. As reported for 3D MAPI and MAPBr [42], the Pb-I bonds, weaker than the Pb-Br ones, may be broken by one or more DMSO molecules acting as Lewis bases, generating different colloidal moieties to which the different dominant textures for Br and I-based films are attributed.

Since no percolation path connects the uppermost microstructures or islands (which show a relatively small filling factor below 10%), any charge transport effect in the thin film plane, the active region of a potential thin film-based device, cannot be assigned to these overlying structures. On the other hand, it is the morphology of the extended (more compact) layer which mostly affects the charge transport and carrier recombination mechanisms, a complete/uniform coverage being required for device application.

### 3.2. Structure and Texture of (PRSH)PbX_3_ Thin Films

#### 3.2.1. Crystal Phase and Compositional Analysis

**Figure 3:**
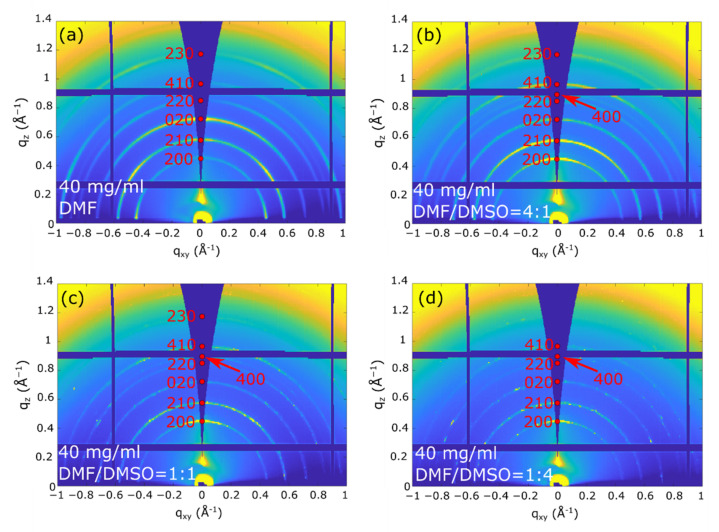
2D-GIXD images taken at 0.2° on the (PRSH)PbBr_3_ films prepared with different DMF/DMSO solvent volume fractions: DMF (**a**), DMF/DMSO = 4:1 (**b**), 1:1 (**c**), and 1:4 (**d**). The reflection indices and the **q** positions of the bulk phase are indicated in red.

**Figure 4:**
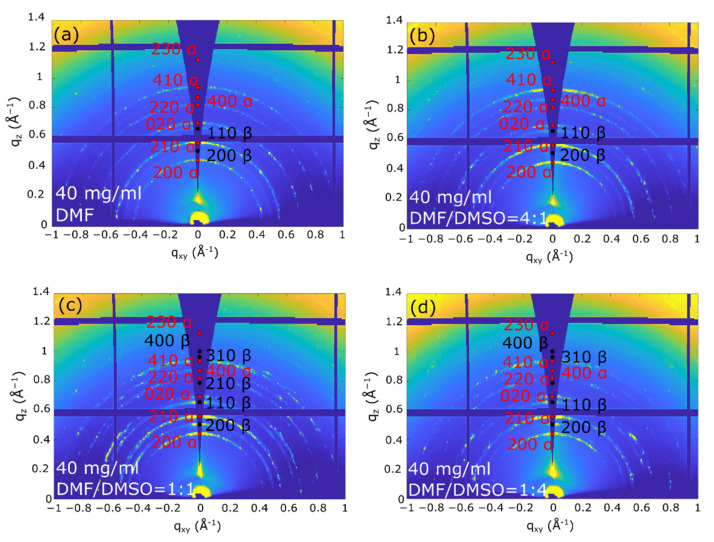
2D-GIXD images taken at 0.2° on the (PRSH)PbI_3_ films prepared with different DMF/DMSO solvent fractions: DMF (**a**), DMF/DMSO = 4:1 (**b**), 1:1 (**c**), and 1:4 (**d**). The reflection indices of the α and β phases and the corresponding **q** positions are indicated in red and black, respectively.

The 2D-GIXD images recorded at α_i_  =  0.2°, where the whole film thickness is probed, (Figure 3 and Figure 4) indicate that all the as-deposited (PRSH)PbX_3_ films are highly crystalline, regardless of the used solvent. The comparison of these 2D images with those collected at α_i_ = 0.05° (see Figures S3 and S4, Supplementary Materials), where the diffracted signals stem only from the uppermost ~4 nm, demonstrates that the film structure is homogeneous across its depth. All the diffraction reflections of the (PRSH)PbBr_3_ films (Figure 3) can be indexed with the known bulk α phase [26], no thin-film phase or residual precursors/contaminants being detected. The indexes and the q positions of the α phase are marked in red in the images. Differently, the 2D-GIXD images of the (PRSH)PbI_3_ films (Figure 4) show the simultaneous presence of the reflections of the two known α and β polymorphic phases (indexes and q positions marked in red and black on the images, respectively). In the bulk, the β phase was previously observed to form at high temperature, but was kinetically stabilized upon cooling to room temperature [26]. The existence of a minor amount of metastable phase at room temperature in spin-coated thin films has been previously reported [46,47]. It is possible that the formation of a minor amount of the β phase can be explained by the much faster crystallization rate during the spin-coating process, as compared to the conventional chemical bench work-up. The %β phase vs. the DMSO/DMF solvent composition was determined by comparing the integrated areas of the pertinent XRD peaks collected in specular geometry, weighted by the corresponding scattering factors. The obtained values are reported in Figure S5, Supplementary Materials. The β phase content, accounting for a few percent when only DMF is used as solvent, increases with the addition of DMSO, peaking at 45% for a DMF/DMSO volume ratio of 1:1, while further increasing the DMSO content results in a reduction of the β phase content to ~15%. Invariance of the relative content of the α and β phases across film thickness was derived from the analysis of the 2D-GIXD images taken at α_i_ = 0.05 and 0.2°. This observation indicates that both the uppermost microcrystals and the underneath layer exhibit, on average, the same α and β crystal phase composition. As discussed below, the β phase content is only marginally affected by the thermal annealing treatment.

As commonly observed for low dimensional perovskites [48,49,50,51], no post-treatment is required to form a crystalline film, and no intermediate composition structures are detected, as opposed to the case of 3D perovskites [44].

We point out that in all the recorded 2D GIXD images (and also in the specular XRD scans presented below), only the hk0 reflections are recorded. Though these reflections possess the largest structure factors, the complete absence of non-zero l signals is here attributed to the tendency of the crystal domains (likely needle-shaped, with the crystallographic c-axis parallel to the longest morphological axis) to align with c* (and c) parallel to the substrate surface (“in plane”). The computed crystal shape determined by using the BFDH model available in the open software Mercury [51], shown in Figure S6, Supplementary Materials, confirms the crystal anisotropy and that the more extended crystal facets are of the hk0 type. Taking into account that, in their crystal structures, all these phases contain 1D chains of face-sharing haloplumbate octahedra running parallel to c, we can easily infer that, in all films, all “inorganic” chains lie flat in the film plane [26].

**Figure 5:**
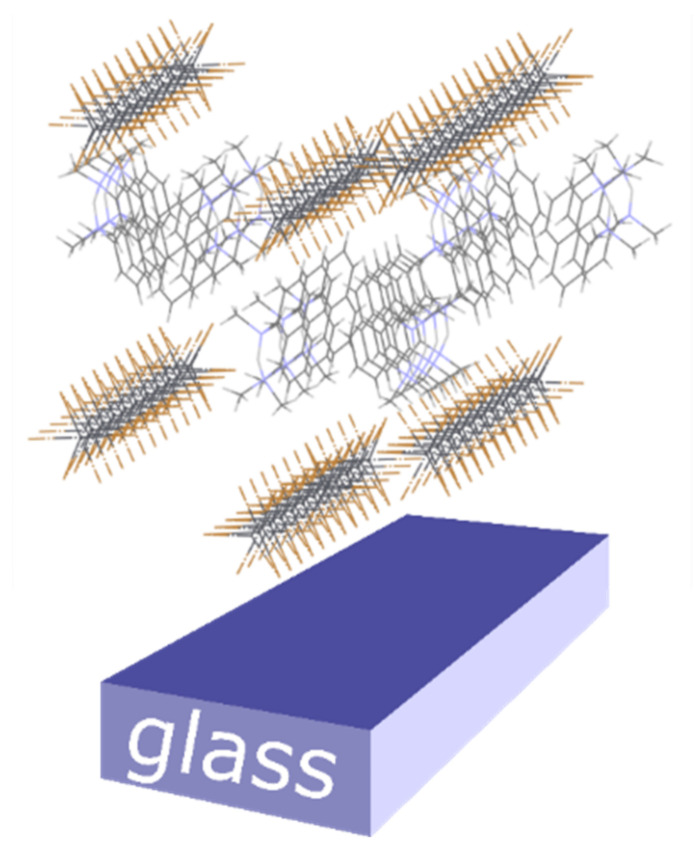
A sketch of the predominant orientation of the 1D chains of (PRSH)PbX_3_, parallel to the substrate surface, as determined by XRD and GIXD.

Additionally, we note in Figure 3 and Figure 4 that all the recorded reflections show enhanced intensity along the q_z_ direction. This differs from what is generally observed in molecular films, where a single family of lattice planes lies parallel to the substrate, i.e., with one preferential orientation of the crystallites [52]. In the present system all the (hk0) planes are preferentially oriented parallel to substrate surface, indicating that all the crystallite orientations giving the 1D octahedral chains aligned parallel to the substrate surface are established (as in the Mikado game), the driving force for crystallite orientation being the 1D chain orientation (see Figure 5). Since the charge transport is expected to preferably take place along these 1D chains, we consider the determined chain orientation highly advantageous for the performance of thin film devices based on parallel configuration.

Nevertheless, not all the crystal orientations are equivalent and the employed solvent (mixture) plays a crucial role in determining the dominant one. This is shown in the detailed analysis of the film texturing reported below.

#### 3.2.2. Texturing

**Figure 6:**
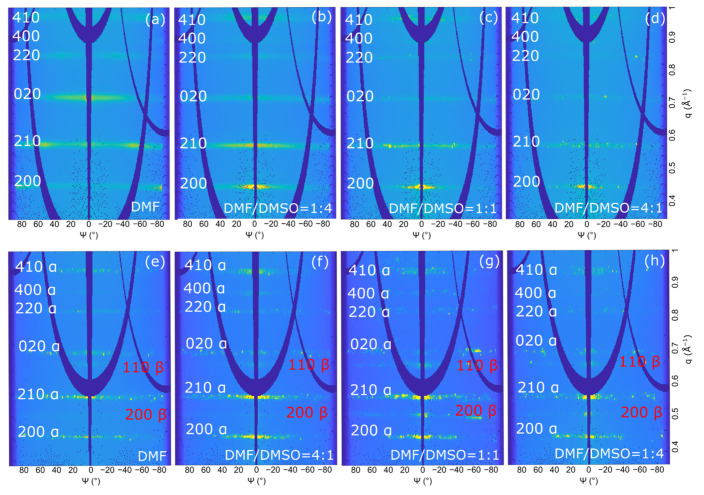
Polar images of the (PRSH)PbBr_3_ (**a**–**d**) and (PRSH)PbI_3_ (**e**–**h**) films prepared with different DMF/DMSO volume ratios, computed starting from the 2D-GIXD images reported in Figure 3 and Figure 4, respectively.

The film texturing can be more easily determined starting from the polar images of the films, obtained by mapping the intensity of the 2D-GIXD images in the (q, ψ) space, where ψ is the azimuthal angle (ψ = 0° indicates perfect alignment between the lattice planes of crystallites and the substrate surface). Figure 6 reports the polar images for the (PRSH)PbBr_3_ and (PRSH)PbI_3_ films as a function of the DMF/DMSO solvent ratio.

For both anions, the texturing significantly changes when DMSO is added to DMF. However, some differences have to be noted. For the (PRSH)PbBr_3_ film prepared from pure DMF, the dominant texturing is 010 [i.e., (010) planes are predominantly oriented parallel to the film surface]; therefrom, the signals of the 210 reflection recorded at ψ = +/− 60° result. When DMSO is added to DMF, the 100 texturing becomes dominant and the 010 texturing is fully lost for DMF/DMSO = 1:1 and 1:4. On the other hand, for (PRSH)PbI_3_ the dominant texturing is the 210 α for the sample prepared with only DMF, to which 100 α texturing is progressively added when binary solvents are used.

These GIXD measurements cannot completely describe the film texturing since, as previously mentioned, the diffraction contributions at q_z_ = 0, i.e., those coming from lattice planes aligned exactly parallel to the substrate surface, cannot be recorded. For this reason, we compared the azimuthally-integrated intensity (AZII, measured along a ring of 2D-GIXD images and generated by lattice planes misaligned with respect to substrate surface) with the specular XRD profiles (diffraction from lattice planes having d* parallel to q_z_).

**Figure 7:**
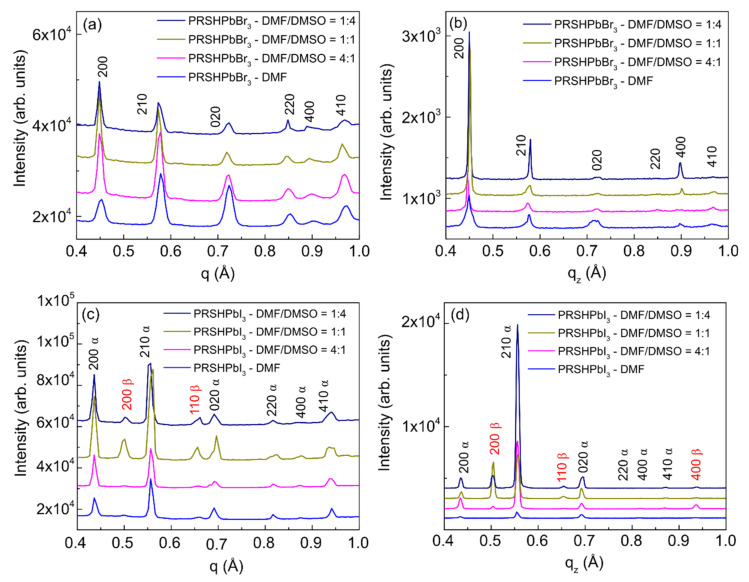
AZII profiles from 2D-GIXD images (**a**,**c**) and specular XRD scans (**b**,**d**) of the (PRSH)PbBr_3_ and (PRSH)PbI_3_ films prepared with different DMF/DMSO volume ratios.

Figure 7 shows the AZII and the corresponding specular scans taken on both the (PRSH)PbBr_3_ (Figure 7a,b) and (PRSH)PbI_3_ (Figure 7c,d) films. As previously observed from the polar images (Figure 6), the specular XRD analysis of the (PRSH)PbBr_3_ films (Figure 7b) reveals that the 100 texturing becomes progressively stronger, and the 010 one much weaker as more DMSO is added in the starting solution. However, undetected by GIXD (Figure 7a), it shows a dominant 100 texturing also for the DMF-only sample. We attribute this difference to the different sample area probed by GIXD (about 0.2 × 5 mm^2^) and specular XRD (about 10 × 5 mm^2^), indicating partial sample inhomogeneity.

The specular XRD analysis carried out on (PRSH)PbI_3_ films confirms that the 210 α texturing is always dominating, and progressively more important as the amount of DMSO in the starting solution increases. It also shows that the 200 α contribution is always very limited. In summary, change of the texturing upon varying the solvent composition is observed for (PRSH)PbBr_3_ films, as reported in the literature for other perovskite-based samples [53], but not for (PRSH)PbI_3_. Nevertheless, for both (PRSH)PbBr_3_ and (PRSH)PbI_3_ samples, the addition of DMSO strengthens the dominant texturing, 100 and 210 α, respectively, a result attributed to the delayed film crystallization caused by the higher basicity of DMSO than DMF, and the subsequent increased ad-atom/ad-molecule mobility. A similar effect was observed for (CH_3_NH_3_PbI_3_) [41]. Determination of film texturing, as here reported, is of paramount importance, as the optical and transport properties of perovskite-based materials were reported to strongly depend on crystal orientation [54].

**Figure 8:**
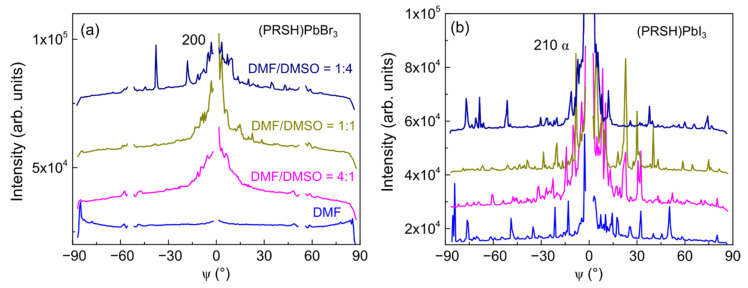
Azimuthal profiles for the dominant 200 reflection of (PRSH)PbBr_3_ (**a**) and 210 α reflection of (PRSH)PbI_3_ (**b**).

As evidenced by the arc-shaped intensity recorded along the Debye rings, a feature more pronounced for the PRSHPbBr_3_ films than for the I-based ones (compare Figure 3 and Figure 4), the crystallites composing the fabricated films have a significant orientational distribution with respect to the preferential one. Mosaicity, defined as the spread of the orientational distribution of the crystallites with respect to substrate normal, is an important structural feature of thin films. A higher mosaicity is indeed commonly associated to a weak(ened) electrical transport performance [55] due to the requirement for charged particles to cross large(er) angle grain boundaries. Mosaicity can be easily visualized in the polar images, being it associated to the dispersion of the diffracted signal, and can be numerically quantified by analyzing the GIXD azimuthal profiles, which, for the dominant 200 reflection of (PRSH)PbBr_3_ and 210 α reflection of (PRSH)PbI_3_, are reported in Figure 8.

Due to the high graininess of the (PRSH)PbI_3_ films (evidenced by the GIXD maps shown in Figure 4), the corresponding azimuthal profiles show a large number of high intensity, but narrow, spikes. In contrast, much smoother profiles are observed for all (PRSH)PbBr_3_ films. These profiles can be matched by summing up a broad and a sharp distribution, corresponding to more randomly oriented and highly textured crystallites, respectively. Though the missing q_xy_ = 0 data partially obscure our analysis, the peak centered at ψ = 0° is always sharper for the (PRSH)PbI_3_ than for the (PRSH)PbBr_3_ sample, indicating a smaller mosaicity in the former, ±5° vs. ±10°, respectively [the 200 peak of the DMF-only sample of (PRSH)PbBr_3_ was excluded from the analysis due to absence of 100 texturing]. Highly comparable values were found upon analyzing the entire set of (PRSH)PbX_3_ films (see Supplementary Materials, Figures S7 and S8).

### 3.3. Effects of Thermal Annealing

#### 3.3.1. *Ex-Situ* Annealing

**Figure 9:**
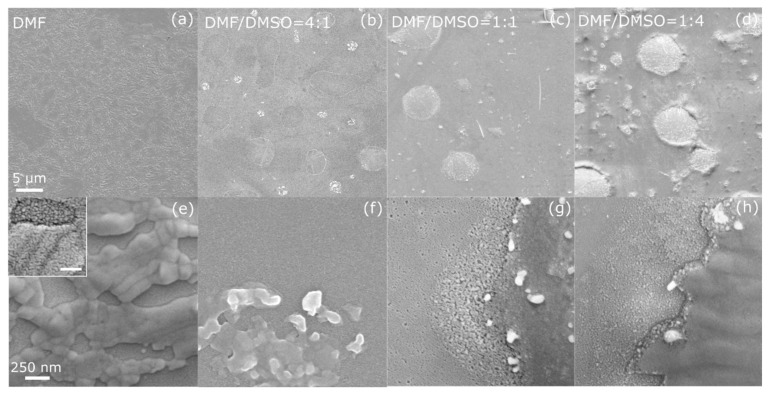
Scanning electron micrographs of annealed (PRSH)PbBr_3_ films prepared in DMF and in DMF/DMSO mixtures, taken at low (**a**–**d**) and high (**e**–**h**) magnification. The scale bars in the left panels apply to all micrographs. The inset in (**e**) shows a high magnification micrograph of the underlying film in (**a**) (the scale bar corresponds to 100 nm).

Although the as-deposited films are already highly crystalline, we investigated the effect of thermal annealing on their morphology and structure. The (PRSH)PbBr_3_/DMF-only film does not show significant morphological modifications after heating in air at 100 °C for 15 min (compare Figure 9 with Figure 1, leftmost panels). At variance, on the surface of the films prepared from DMF/DMSO solvent mixtures (Figure 9f–h), crystallites up to 250 nm in length are formed by annealing, mostly confined within the large micrometric islands present at substrate surface.

**Figure 10:**
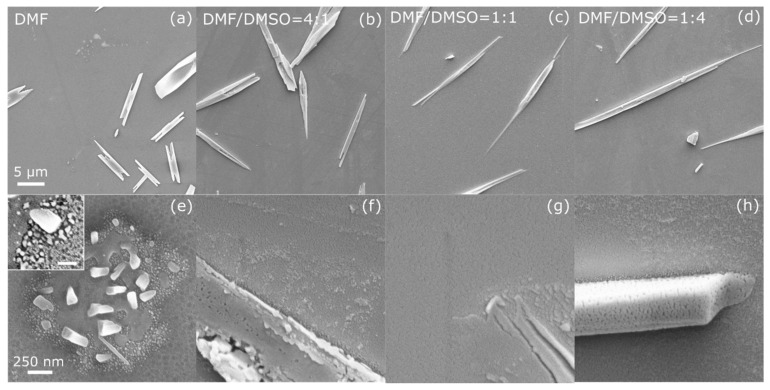
Scanning electron micrographs of annealed (PRSH)PbI_3_ films prepared in DMF and in DMF/DMSO mixtures, taken at low (**a**–**d**) and high (**e**–**h**) magnification. The scale bars in the left panels apply to all micrographs. The inset in (**e**) shows a high magnification micrograph of the film in (**a**) (the scale bar corresponds to 100 nm).

Differently, the (PRSH)PbI_3_/DMF-only film shows a considerable reorganization after annealing (Figure 10e), with small grains merging into larger and elongated crystallites, up to 250 nm long. All other films prepared from DMF/DMSO solutions are insensitive to heat treatment (Figure 10f–h), apart from showing a minor desorption, leading to porous, spongy microcrystals (see Figure 10h).

In agreement with the minimal morphological evolution, the substantial invariance of the XRD scans recorded before and after annealing (Figure S9, Supplementary Materials) indicates that the structure, texturing, and thickness of the (PRSH)PbX_3_ films are not significantly altered by the thermal treatment. The only evident effect is the occasional decrease of the diffracted intensity, suggesting a limited film degradation at 100 °C, in agreement with desorption of a minor amount of material observed in Figure 10h.

The film thermal stability is further investigated on representative (PRSH)PbX_3_ samples, comparing the specular XRD patterns and the absorption spectra recorded after annealing at 100 °C for longer times, for up to 60 min (Figure S10). For the (PRSH)PbBr_3_ film, annealing does not produce significant changes either in the XRD patterns (Figure S10a) or in the absorption spectrum (Figure S10c). These results indicate the thermal stability of the (PRSH)PbBr_3_ film. For the (PRSH)PbI_3_ film, on the other hand, annealing induces a significant decrease in the intensity of the diffracted peaks, already after 30 min (Figure S10b), together with a weak, approximately constant decrease in light absorption (Figure S10d). The absence of novel absorption signals again allows us to exclude the formation of by-products, in contrast with what previously reported for 3D hybrid perovskites like MAPI [56]. All these data, together with the SEM images of the films annealed for 15 min (see Figure 10h), strongly suggest that annealing only induces desorption of the (PRSH)PbI_3_ film without the formation of amorphous secondary compounds.

As anticipated, in the (PRSH)PbI_3_ films the variation in the β phase content upon annealing for all the different solvent mixtures is weak or null (Figure S5).

Scherrer’s equation is used to determine the average crystallite size before and after annealing for the different samples and crystal orientation (Figures S11 and S12, Supplementary Materials). Although the crystallite size obtained from Scherrer’s equation seems to be inconsistent with the SEM data showing 5/10 nm-large grains at film surface (see Figures S1a–d and S2a–d), XRD analysis provide a mass-distribution-based average value, with a minimal contribution from small crystallites, and a larger contribution from larger crystals in the film (lying at the film/substrate interface or within the islands/microcrystals at film surface). Figures S11 and S12 show an additional peak broadening, which suggests a reduction of the average size of coherently diffracting domains upon annealing.

Importantly, no additional crystalline sub-products are observed as a result of weak film degradation, a problem often encountered in 3D perovskite counterparts [57]. As for both set of films degradation is not observed, we consider these materials suitable for direct deposition or printing on low softening point bendable substrates, toward they use in flexible electronics applications.

#### 3.3.2. *In-Situ* Annealing

Although ex-situ annealing experiments did not show any improvement in the crystalline quality of the films, in-situ synchrotron GIXD measurements were carried out, during annealing, to investigate any possible effect of the thermal process, even if small and reversible, such as expansion/contraction and α to β phase transition. Taken as representative for the (PRSH)PbI_3_ set of samples, the one deposited using DMF-only was studied.

Figure S13 reports the sequence of the 2D-GIXD images collected, one every 10 min, on the film kept at the constant temperature of 130 °C. Significantly, the q positions of the different reflections evolve during annealing, moving from the theoretical bulk values, found in the pristine film, towards higher values, with the exception of the reflection 200 β, whose q value slightly lowers. These observations indicate that a contraction of all (measurable) lattice planes occurs upon annealing (but an expansion for the 200 β ones), i.e., that a homogeneous strain is introduced at high temperature. As the entire Debye-Scherrer rings move to larger (lower for the 200 β reflection) q values, this indicates that the thermal treatment results in hydrostatic strain applied to the crystallites, regardless their orientation on the surface. The decrease in lattice constant cannot be ascribed to the solvent desorption since, as already mentioned, the lattice constants of the as-deposited film are those of the bulk crystal, where no solvent molecules are included. The most plausible explanation for the introduction of hydrostatic strain is surface stress. Likely driven by the observed reduction of the crystallite size (Figure S12) and by the consequent enhancement of the surface to volume ratio, the observed hydrostatic strain is a consequence of the occurrence of surface (tension) effects. Similar effects were previously reported for other metallic and semiconducting nanocrystalline systems [58,59,60], which showed surface-stress induced homogeneous strain for sufficiently small nanoparticle sizes. Looking carefully at the sequence of the 2D GIXD images reported in Figure S13, we verified that, for each reflection, spots corresponding to different crystallites change their q’s at different times, leading to a spread of the q values recorded in each image. This mimics an inhomogeneous strain within the film and results in broadening of the diffraction peaks, which cannot be attributed to crystallite size reduction only.

**Figure 11:**
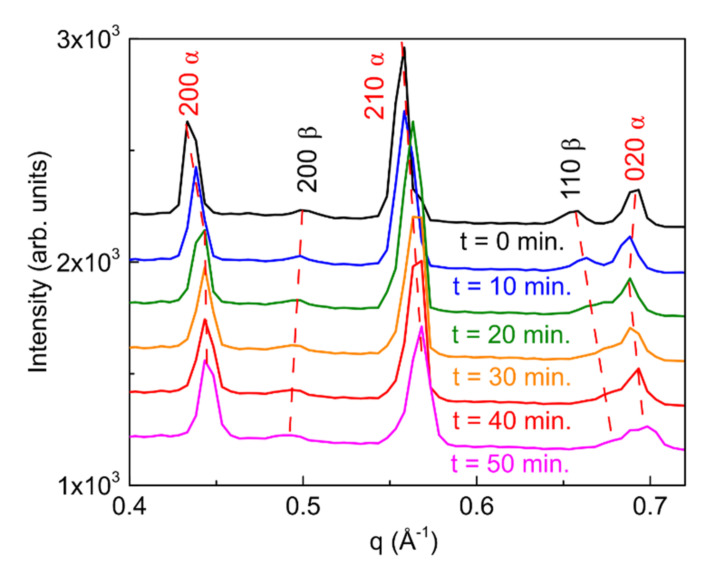
Integrated scans along the Debye-Scherrer rings obtained from selected 2D-GIXD images collected during in-situ annealing for the (PRSH)PbI_3_ DMF-only sample investigated in Figure S12. The dotted lines are guides to the eyes highlighting the shift of the diffraction peaks upon annealing.

To better visualize and quantify the structural evolution of the film upon annealing, the integrated scans along the Debye-Scherrer rings of the 2D-GIXD images shown in Figure S13 are reported in Figure 11. After 50 min of annealing, variations of q of +2.2%, −2.1%, +1.9% and +1.2% are determined for the 200 α, 200 β, 210 α and 020 α reflections, respectively. We note that the rate of q variation upon annealing depends on the specific reflection and on the phase, indicating a different reactivity to temperature for the different sets of crystallographic planes, possibly due to the different ending molecules in the crystallographic planes under analysis.

Upon annealing, in addition to the mentioned shifts of the diffraction peaks, we also observe peak broadening (see e.g., the 020 α contribution in Figure 11), which was already commented and which is consistent with the results of ex-situ annealing experiments (Figure S9).

### 3.4. Effect of Film Aging

To study the time stability of the annealed (PRSH)PbX_3_ films, these were investigated by XRD after seven months aging, being kept in the dark under a weak N_2_ stream (see Figure S14, Supplementary Materials). For all samples, aging does not result in the formation of by-products. Though some significant intensity drops were occasionally observed for some samples, no PbO peaks were found, and, in some cases (Figure S14a,b,e), no variation in the recorded XRD traces was observed at all. Some (expected) material desorption is observed at the SEM for the annealed samples but not for aged ones. For this reason, we attribute the decrease in the diffracted intensities, which is similar for the heated and aged films, to material desorption in the former case, and mainly to lowering of the film crystallinity for the latter case. Experimentally, we did not observe any change in the XRD background height and shape, suggesting that no significant amounts of amorphous phases are formed upon aging. However, the presence of traces of amorphous PbO cannot be excluded. As for thermal annealing, also aging did not influence the film thickness.

In summary, these results indicate that, if suitably deposited, (PRSH)PbX_3_ films may exhibit excellent stability in time even without the presence of any capping/passivation layer. This aspect makes them nearly comparable to state-of-the-art 2D perovskite thin films [18,61] and better than 3D perovskites [62].

### 3.5. Effect of UV Light Exposure

The structural stability to UV light of two representative (PRSH)PbX_3_ films (those prepared in DMF) was investigated by studying the evolution of specular XRD profiles during sample irradiation with a power density of 600 mW/cm^2^, using an LED emitting at λ = 380 nm. For both films no changes in the collected diffractograms are observed after up to 15 h of UV exposition. The XRD patterns recorded before and after 15 h of illumination are reported in Figure S15, Supplementary Materials. This result is in agreement with previous ones showing a high stability against UV radiation of MAPBr_3_ [56,63] and of several 1D and 2D perovskite systems [64,65,66]. Less common is the pronounced structural stability of iodine-based counterparts [56,63]. Moreover, the obtained result confirms our previous evidence of the highly stability against short wavelength radiation of mixed anion 1D (PRSH)PbI_2_Br and (PRSH)PbIBr_2_ powders [26], which is rather unusual for 3D hybrid mixed perovskites [56].

### 3.6. Stability of Thin Films in a Controlled Humidity Environment

The stability of the fabricated films against humidity was investigated by following the evolution of their XRD profiles, taken on the bare aged films and on the same films after sequential exposition to different levels of relative humidity (RH). The XRD scans taken on the (PRSH)PbBr_3_ films at different humidity conditions are reported in Figure S16. As evident from the reported diffractograms there is no constant response of the film microstructure when exposed to high RH levels. While for the DMF-only sample and the one with DMF/DMSO = 1:1 the recorded diffractograms do not show film degradation after 25 days at RH = 78%, and actually the intensity of the 200 and 210 peaks increases, suggesting further humidity-induced film crystallization, the samples prepared with excess DMF and excess DMSO in mixed solvents degrade. For the latter, severe reorganization of the crystals is also observed before complete degradation, with texturing moving from 200 to 210 (Figure S16d). A very low intensity (still uninterpreted) peak appears at 2θ ~9.6°, which cannot be attributed to any PbO polymorph or to any known other oxo/hydrated crystal form. Whether it originates from sample hydrolysis needs further investigation. A similar situation is observed for (PRSH)PbI_3_ films (see Figure S17), but in this case all samples show a significant reduction of the diffraction peaks intensities upon exposition to RH = 78% for 25 days. Moreover, for the sample prepared with excess DMF (Figure S17b), a PbI_2_-related peak appears after 25 days at RH = 78%.

In summary, the behavior of (PRSH)PbX_3_ films under high RH conditions heavily depends on the used solvent, RH-assisted crystallization (similar to what previously reported for 0D perovskites under high humidity conditions [67]), partial film decomposition, or PbI_2_ formation being observed. Using the appropriate solvent (mixture), (PRSH)PbBr_3_ films exhibit not only excellent time and UV radiation stability, but also very good stability against high humidity levels, with only minor variation in the recorded diffractograms, making them suitable in high-humidity applications. The better stability against humidity of the Br-containing films, as compared to the I-based ones, is in agreement with previous observations on 3D perovskite systems, where the enhanced stability of MAPbBr_3,_ compared to that of MAPbI_3_, was attributed to suppression of organic cation migration and to the slow(er) bromide migration rate [68]. Nevertheless, the non-monotonic trend of film stability vs solvent composition raises an additional warning, for an issue that is still to be clarified.

## 4. Conclusions

We have here investigated the morphology, texturing, structure, as well as thermal and environmental stability of thin films based on newly developed 1D (PRSH)PbBr_3_ and (PRSH)PbI_3_ pseudo-perovskites. We have found that the morphology of the synthesized films heavily depends on the halide and, to a lower extent, on the DMF/DMSO solvent ratio. All the as-prepared films are highly crystalline without the need of any high temperature treatment, and thus they are compatible with low softening point flexible substrates. They also show the same solid phase of the bulk materials and do not contain other crystalline contaminants. The texturing of the prepared films and, in the case of (PRSH)PbI_3_ films, the fraction of the β phase as well, can be modified by adding DMSO to DMF in different concentrations. Our combined specular XRD and GIXD analyses indicate that the 1D chains of face-sharing haloplumbate octahedra present in the (PRSH)PbX_3_ materials always lie parallel to the substrate surface, a result that is expected to have profound consequences on the charge transport effects in these films, promoting anisotropic transport. Thermal annealing at 100 °C has no beneficial effect on the film crystallinity, resulting in a partial decrease in the diffracted intensity (the magnitude of which depends on the film preparation conditions). Films of the Br-containing compounds have shown extreme stability in time, when kept under a N_2_ stream and in the dark. In addition, exposition to UV radiation of representative films has shown no structural changes in the diffraction pattern after up to 15 h at an incident power of 600 mW/cm^2^. Finally, the film response to humidity is found to depend on the DMF/DMSO volume ratio, with the best samples showing nearly complete humidity tolerance, as determined by only minor variations in the recorded diffractograms, or even humidity induced crystallization after up to 25 days at a RH value of 78% without any passivation/capping layer. The obtained results, together with the large material bandgap in the near UV range [26], make the developed thin films promising as capping layers for perovskite-based solar cells and for other electronic applications such as thermoelectrics.

## Data Availability

The data presented in this study are available on request from the corresponding author.

## Supplementary Materials

The following are available online at https://www.mdpi.com/article/10.3390/nano11102765/s1. Figure S1: High magnification plane-view and low magnification cross-sectional scanning electron micrographs taken on (PRSH)PbBr_3_ films prepared with a powder concentration of 40 mg/mL in DMF and in mixed DMF/DMSO solvents with a volume ratio of 4:1, 1:1 and 1:4; Figure S2: High magnification scanning electron micrographs taken on (PRSH)PbI_3_ films prepared with a powder concentration of 40 mg/mL in DM and in mixed DMF/DMSO solvents with a volume ratio of 4:1, 1:1 and 1:4; Figure S3: 2D-GIXD images taken at 0.05° on (PRSH)PbBr_3_ films as a function of the DMF/DMSO solvent volume ratio; Figure S4: 2D-GIXD images taken at 0.05° on (PRSH)PbBr_3_ films as a function of the DMF/DMSO solvent volume ratio; Figure S5: Evolution of the β phase percentage in the film as a function of the DMSO % in the starting solution before and after annealing; Figure S6: Theoretical crystal morphology for (PRSH)PbX_3_, computed using the BFDH model available in Mercury; Figure S7: Mosaicity of (PRSH)PbBr_3_ films for the 210 and 020 crystal planes, as a function of the exploited solvent; Figure S8: Mosaicity of (PRSH)PbI_3_ films for the 200 α, 020 α, 200 β, and 110 β crystal planes, as a function of the exploited solvent; Figure S9: XRD θ/2θ scans for the (PRSH)PbBr_3_ and (PRSH)PbI_3_ films taken before and after annealing, as a function of the exploited solvent; Figure S10: XRD θ/2θ scans for the DMF-only (PRSH)PbBr3 and (PRSH)PbI3 films taken under different annealing conditions and transmittance and absorption spectra of the same films. Figure S11: Bottom bound for the mean crystallite size of 200-, 210-, and 020-oriented crystallites in (PRSH)PbBr_3_ films before and after annealing, as a function of %DMSO in DMF; Figure S12: Bottom bound for the mean crystallite size of 200 α-, 210 α-, 020 α- and 110 β -oriented crystallites in (PRSH)PbI_3_ films before and after annealing, as a function of %DMSO in DMF.; Figure S13: 2D-GIXD images of the DMF-only (PRSH)PbI_3_ film taken in-situ after 0, 10, 20, 30, 40, and 50 min while heating the sample to the constant temperature of 130 °C; Video 1: in-situ GIXD (PRSH)PbI_3_: Corresponding video reporting the series of the 2D-GIXD images recorded during in-situ heating at 130 °C. The images are recorded every 90 sec.; Figure S14: XRD θ/2θ scans of annealed (PRSH)PbBr_3_ and (PRSH)PbI_3_ films taken before and after aging, as a function of the exploited solvent; Figure S15: Specular XRD patterns for the aged DMF-only (PRSH)PbBr_3_ (a) and (PRSH)PbI_3_ (b) films taken prior and after exposition to UV light for 15 h (λ = 380 nm, power density = 600 mW/cm2); Figure S16: XRD θ/2θ scans taken on the aged (PRSH)PbBr_3_ films after sequential exposition to 44% relative humidity for 2 days, and to 78% relative humidity for 1, 5, and 25 days; Figure S17: XRD θ/2θ scans taken on the aged (PRSH)PbI_3_ films after sequential exposition to 44% relative humidity for 2 days, and to 78% relative humidity for 1, 5, and 25 days.
