# Supplementary material for: Highly Stable Thin Films Based on Novel Hybrid 1D (PRSH)PbX3 Pseudo-Perovskites
Source: Nanomaterials (Basel). 2021 Oct 19;11(10):2765. doi: 10.3390/nano11102765 (PMC8539398; doi:10.3390/nano11102765)
Supplement: Supplementary file 1 [file nanomaterials-11-02765-s001.zip › nanomaterials-1381140-SI.pdf]

Supplementary information for manuscript

Highly Stable Thin Films Based on Novel Hybrid 1D $(PRSH)PbX_3$ Pseudo-Perovskites

Gabriele Calabrese [1,*], Candida Pipitone [2], Diego Marini [1], Francesco Giannici [2], Antonino Martorana [2], Luisa Barba [3], Caterina Summonte [1], Norberto Masciocchi [4,*] and Silvia Milita [1,*]

[1] Istituto per la Microelettronica e Microsistemi, Consiglio Nazionale delle Ricerche, via Gobetti 101, 40129 Bologna, Italy; marini@bo.imm.cnr.it (D.M.); summonte@bo.imm.cnr.it (C.S.)
[2] Dipartimento di Fisica e Chimica, Università di Palermo, viale delle Scienze, Ed. 17, 90128 Palermo, Italy; candidapipitone@gmail.com (C.P.); francesco.giannici@unipa.it (F.G.); antonino.martorana@unipa.it (A.M.)
[3] Istituto di Cristallografia, Consiglio Nazionale delle Ricerche, Strada Statale 14-km 163, 5, AREA Science Park, Basovizza, 34149 Trieste, Italy; luisa.barba@ic.cnr.it
[4] Dipartimento di Scienza e Alta Tecnologia e To.Sca.Lab., Università dell'Insubria, via Valleggio 11, 22100 Como, Italy
* Correspondence: calabrese@bo.imm.cnr.it (G.C.); norberto.masciocchi@uninsubria.it (N.M.); milita@bo.imm.cnr.it (S.M.)

Index:

1. **Additional SEM images of $(PRSH)PbX_3$ films**
2. **2D-GIXD maps taken on $(PRSH)PbX_3$ films at 0.05°**
3. **Phase composition of the $(PRSH)PbI_3$ films**
4. **Theoretical crystal morphology**
5. **Mosaicity of $(PRSH)PbX_3$ films**
6. ***Ex-situ* XRD and Optical measurements of annealed $(PRSH)PbX_3$ films**
7. **Vertical dimension of the coherently diffracting domains in $(PRSH)PbX_3$ films**
8. ***In-situ* 2D-GIXD mapes recorded during thermal annealing**
9. **Specular XRD patterns of $(PRSH)PbX_3$ films before and after aging**
10. **Specular XRD patterns of $(PRSH)PbX_3$ films under exposition to UV light**
11. **Specular XRD patterns of $(PRSH)PbX_3$ films under different relative humidity conditions**

1. Additional SEM images of $(PRSH)PbX_3$ films

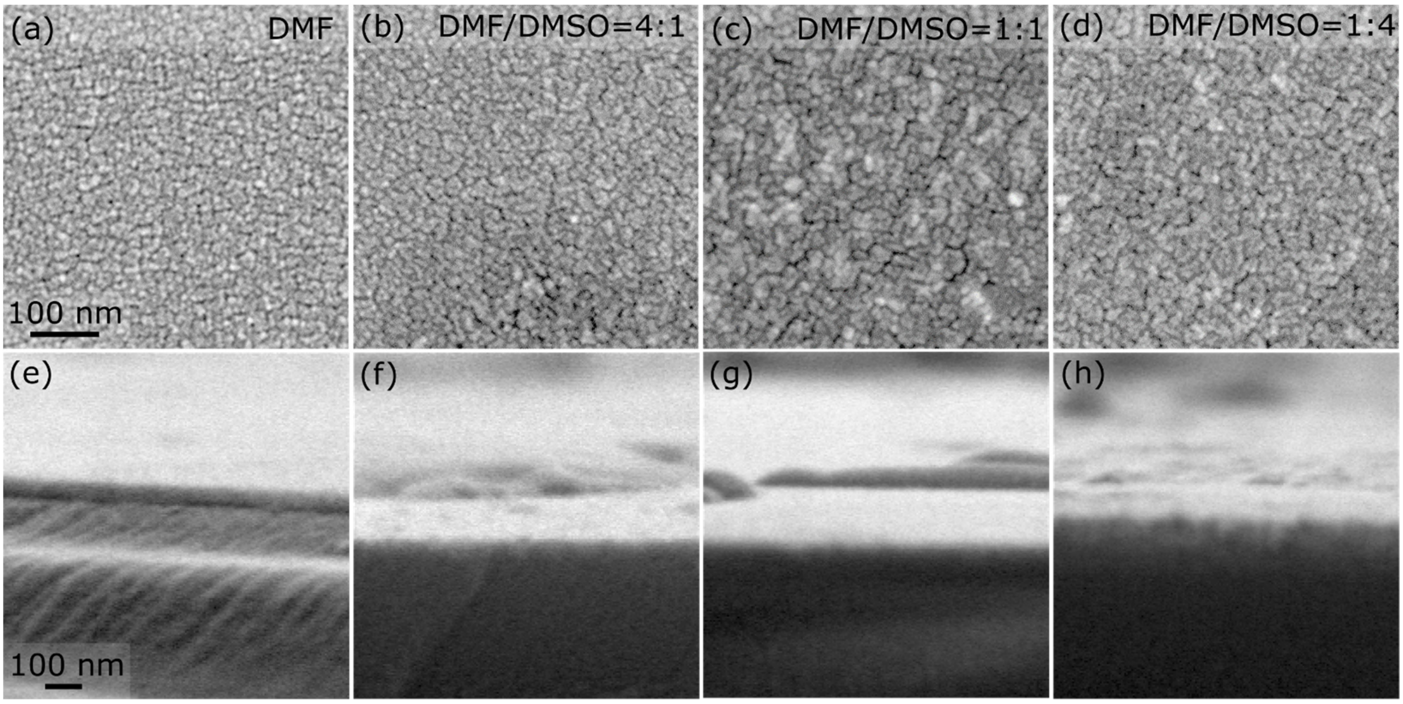

Figure S1. (a) – (d) High-magnification top-view SEM images of $(PRSH)PbBr_3$ films prepared in DMF and in mixed DMF/DMSO solvents with a volume ratio of 4:1, 1:1, and 1:4. (e) – (h) Low magnification cross-sectional scanning electron micrographs of the same films investigated in (a) – (d).

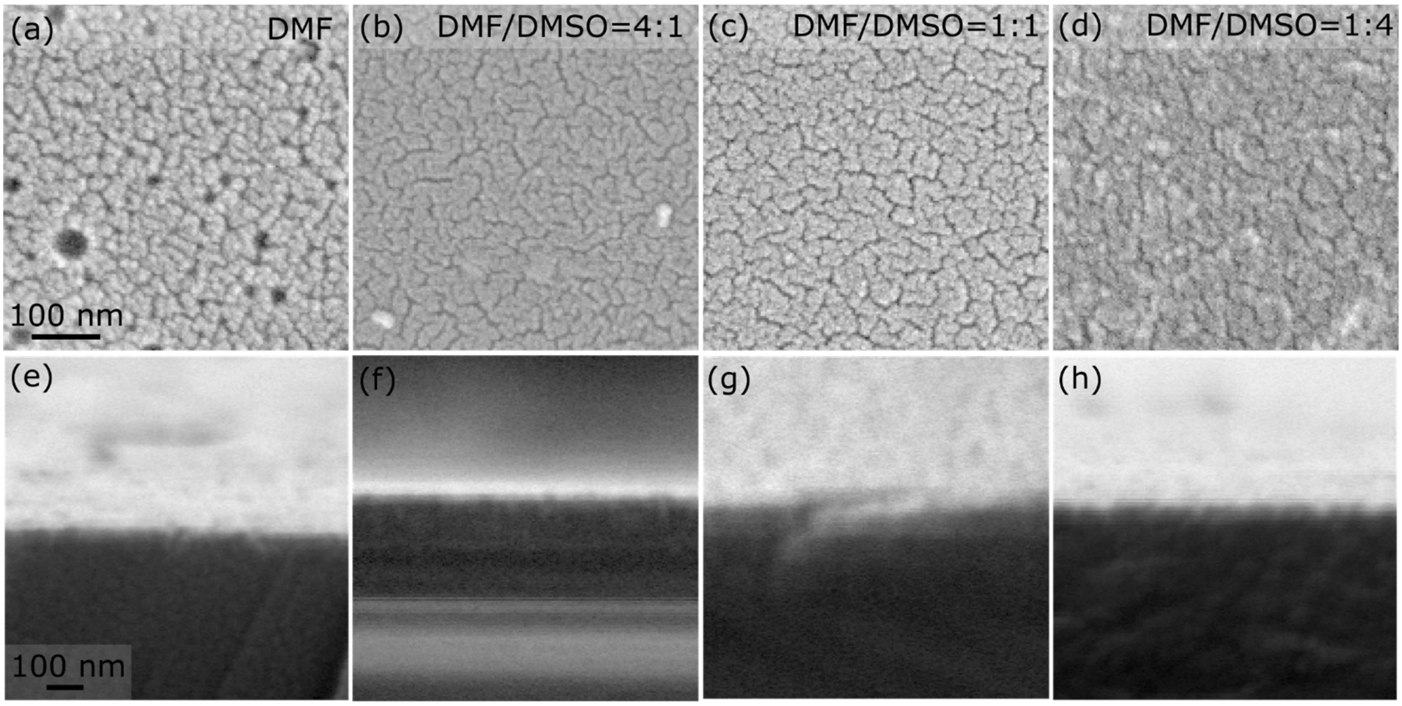

Figure S2. (a) – (d) High-magnification top-view SEM images of $(PRSH)PbI_3$ films prepared in DMF and in mixed DMF/DMSO solvents with a volume ratio of 4:1, 1:1, and 1:4. (e) – (h) Low magnification cross-sectional scanning electron micrographs of the same films investigated in (a) – (d).

2. 2D-GIXD maps taken on (PRSH)PbX$_3$ films at 0.05°

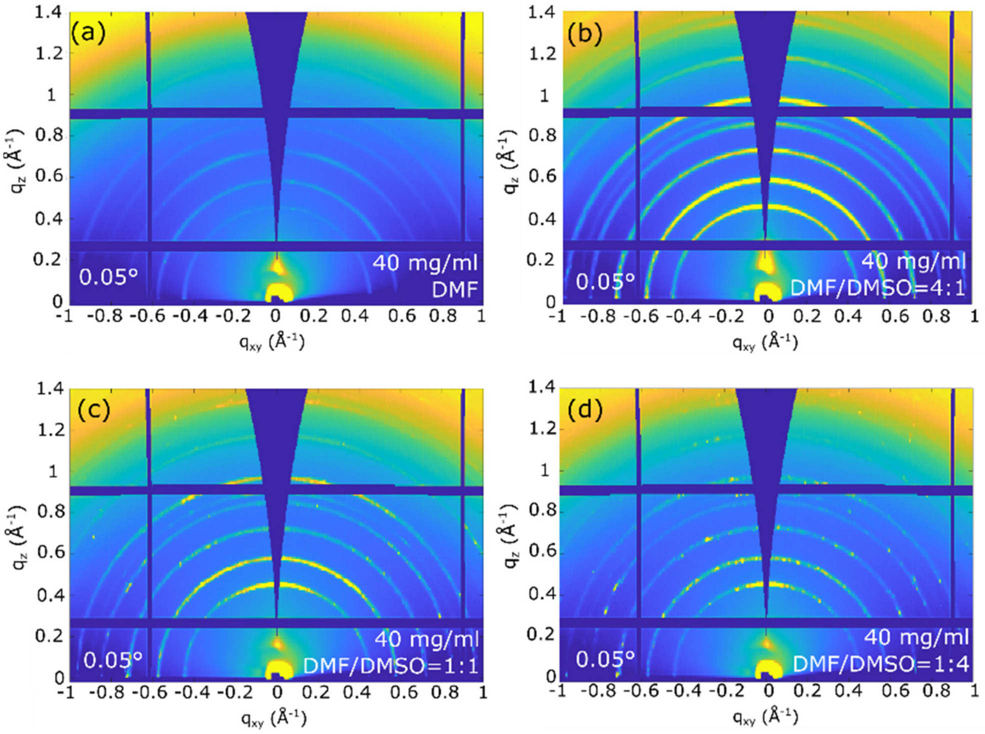

Figure S3. 2D-GIXD images taken at 0.05° on (PRSH)PbBr$_3$ films as a function of the DMF/DMSO solvent volume ratio.

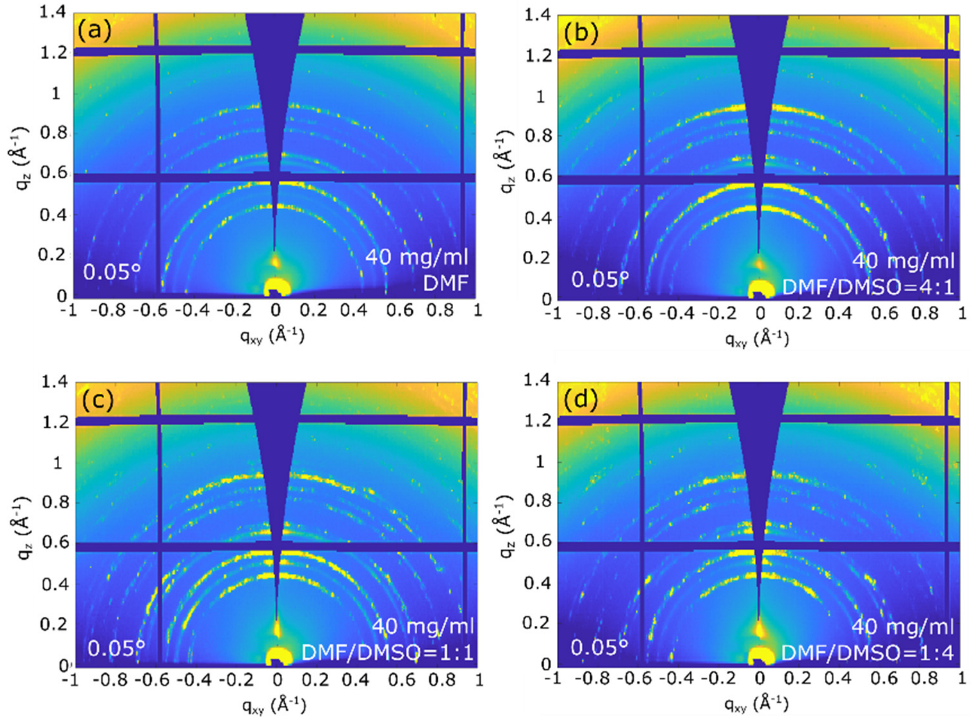

Figure S4. 2D-GIXD images taken at 0.05° on (PRSH)PbI$_3$ films as a function of the DMF/DMSO solvent volume ratio.

3. Phase composition of the (PRSH)PbI_3 films before and after annealing

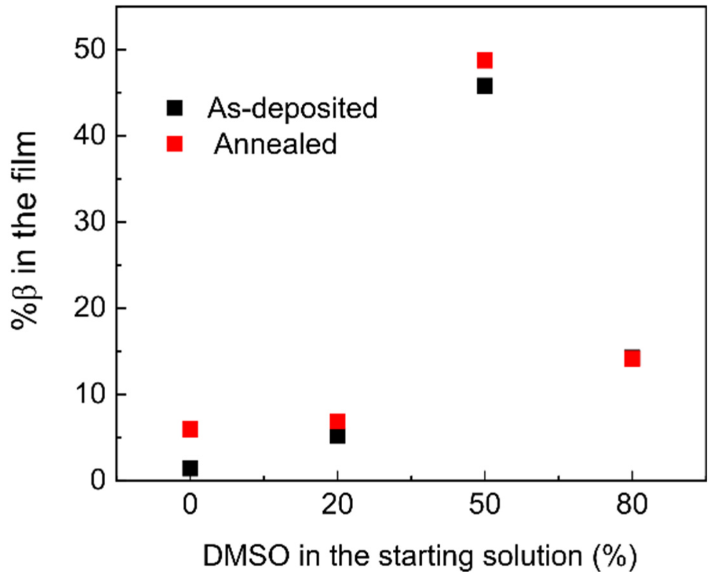

Figure S5. Evolution of the β phase percentage in the film as a function of the DMSO % in the starting solution before (black points) and after annealing (red points).

4. Theoretical crystal morphology

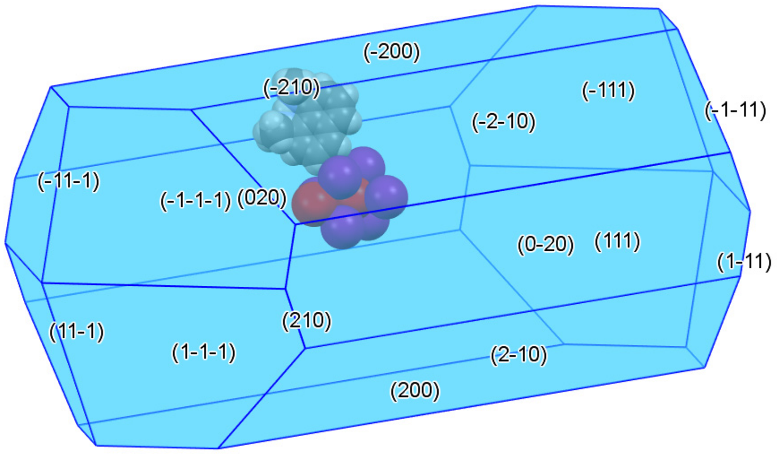

Figure S6. Theoretical crystal morphology for (PRSH)PbX_3, computed using the BFDH model available in Mercury software [1].

5. Mosaicity of (PRSH)PbX3 films

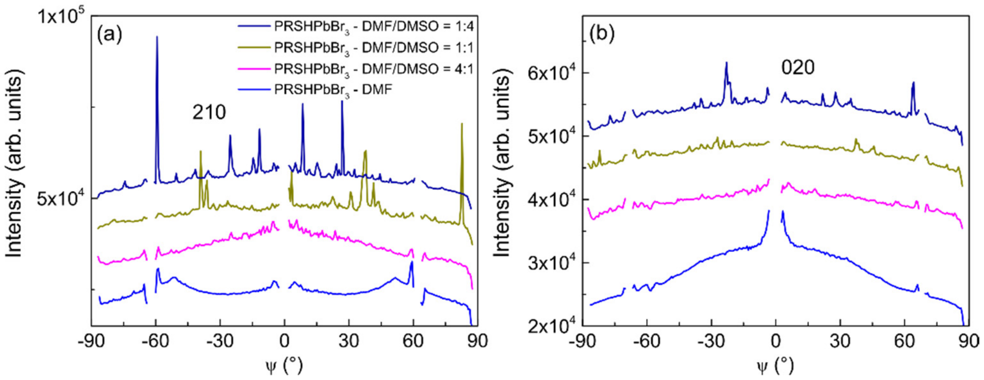

Figure S7. Azimuthal profiles of (PRSH)PbBr3 films for the 210 (a) and 020 (b) crystal planes, as a function of the used solvent.

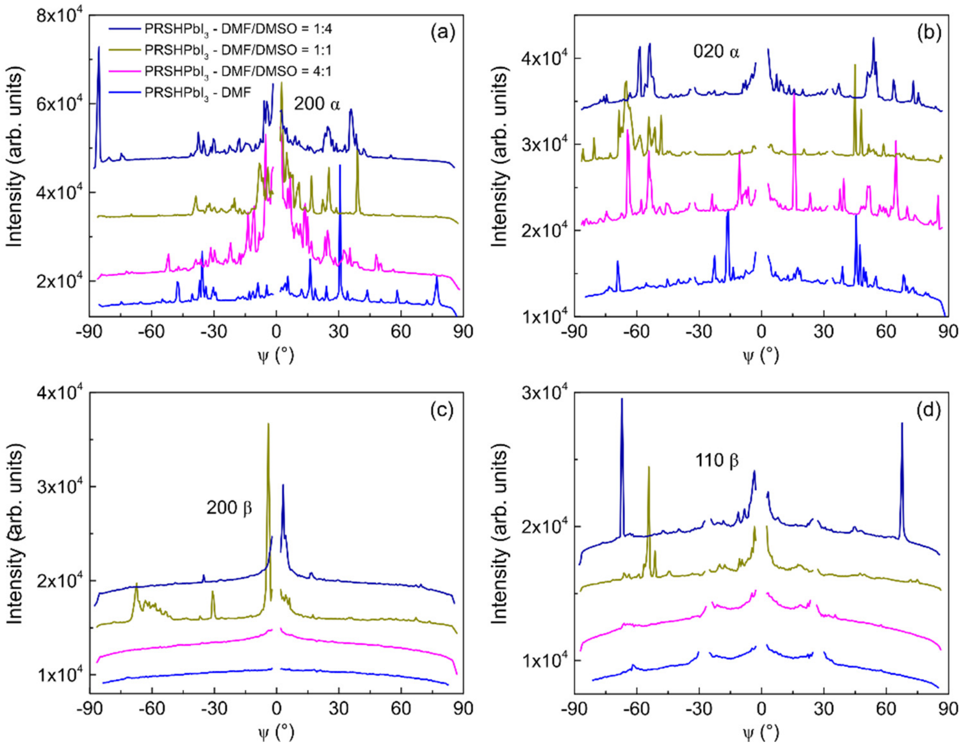

Figure S8. Azimuthal profiles of (PRSH)PbI3 films for the 200 α (a), 020 α (b), 200 β (c), and 110 β (d) crystal planes, as a function of the used solvent.

The mosaicity is given by the HWHM (half width at half maximum) of the azimuthal profiles.

6. *Ex-situ* XRD and Optical measurements of annealed (PRSH)PbX3 films

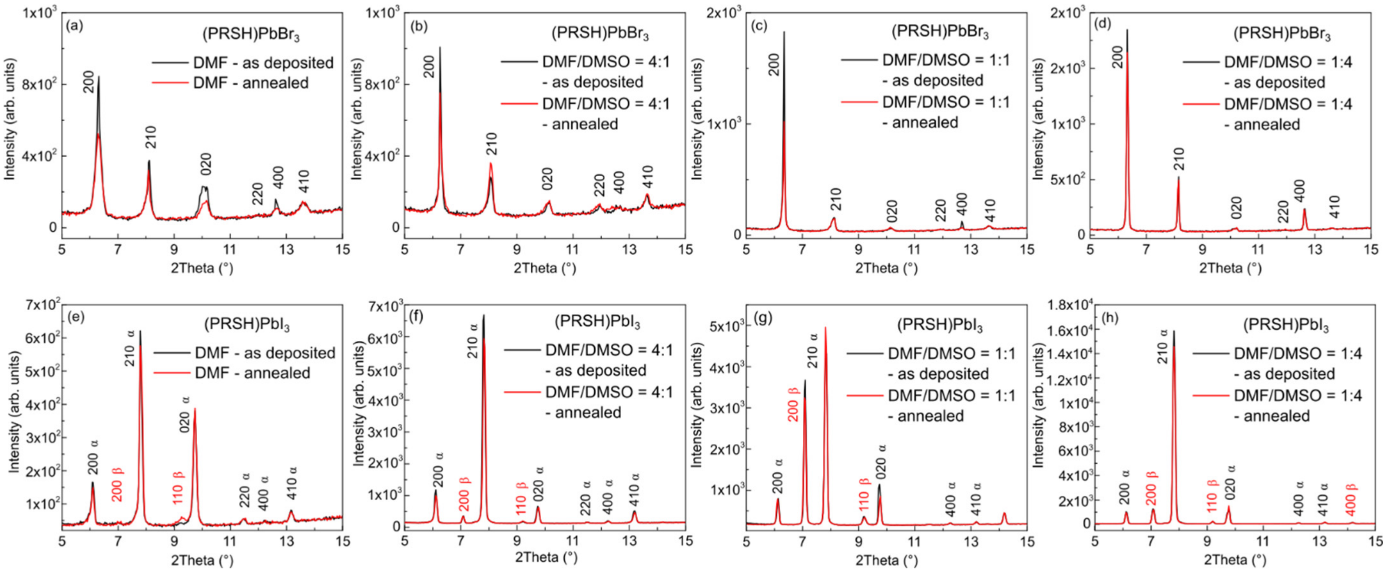

Figure S9. Specular XRD patterns for the (PRSH)PbBr3 (a-d) and (PRSH)PbI3 (e-h) films taken before (black) and after (red) annealing at 100 °C for 15 min,for the different used solvent.

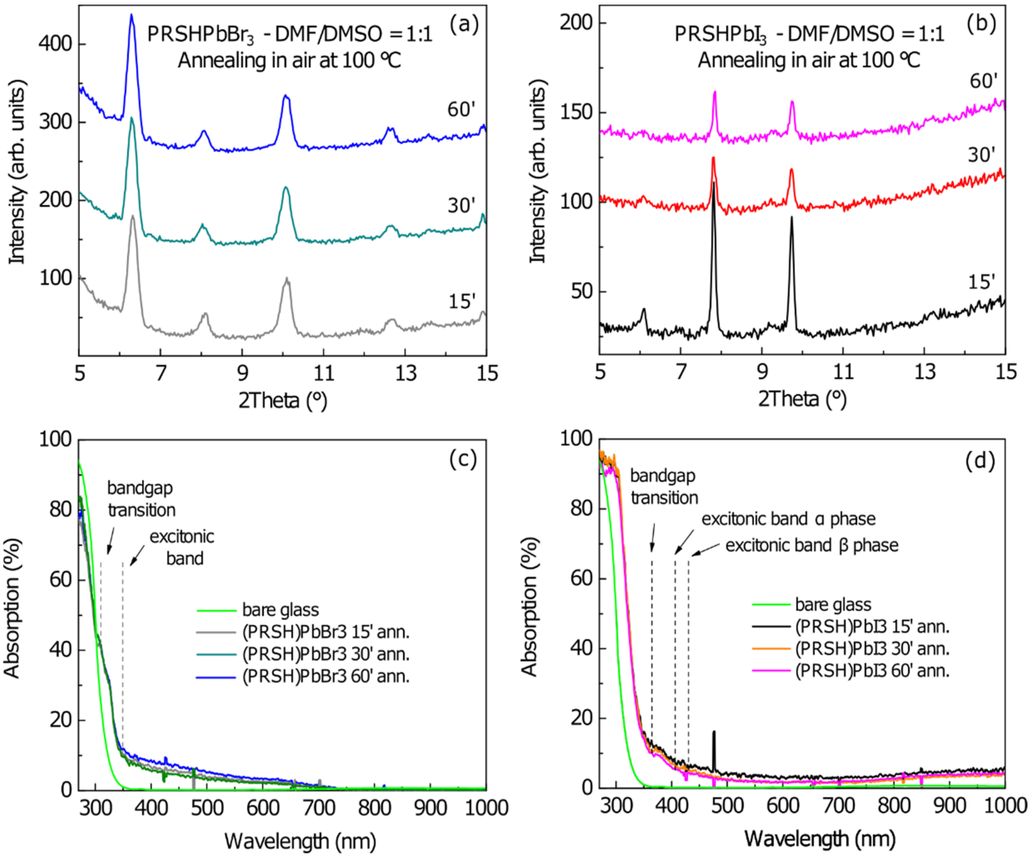

Figure S10. Specular XRD patterns and sphere-integrated absorption spectra for the DMF-only (PRSH)PbBr3 (a and c) and (PRSH)PbI3 (b and d) films taken after annealing at 100 °C for 15, 30 and 60 minutes. The absorption curve of the substrate is reported in light green. The *dotted lines in c and d correspond to the peak positions of the absorption spectra of the corresponding powder sample [26].*

The Absorption spectra of the (PRSH)PbX3 films (reported in Figs. S9 c and d) are obtained using the equation A(%) = 100-T(%)-R(%), where T(%) and R(%) are the transmittance and reflectance of the sample. As a reference, the absorption spectrum of the bare glass substrate has been also reported (light green curve in Figs. S9 c and d). The lower signal-to-noise ratio for the film

spectra is due to the use of the integrating sphere (not used in the characterization of the glass substrate) and to the associated much lower signal.

7. Vertical dimension of the coherently diffracting domains in (PRSH)PbX_3 films

From the recorded specular XRD patterins it is also possible to estimate, from the FWHM of the diffraction peaks, the average size of the coherently diffracting domains, i.e. the vertical correlation length L^v. The values of L^v are determined by using the Scherrer's equation under the assumption of Gaussian intensity distributions and neglecting the instrumental and strain broadening. While the instrumental contribution to line broadening is at least 20 times smaller than the recorded linewidths on bulk substrates using the same experimental setup, line broadening due to inhomogeneous strain is hard to quantify for the samples under investigation. It follows that the obtained values of L^v represent a bottom bound for the size of the coherently diffracting domains in the studied films.

The values of L^v determined for the dominant contributions of the (PRSH)$PbBr_3$ films are reported as a function of %DMSO in **Figure S11**. With the addition of DMSO, L^v increases for all reflections, but its behaviour is not-monotonic, the smallest value is always obtained for DMF-only solvent and is in the range of 27-40 nm. For most reflections the thermal annealing induces a reduction in L^v, a result that is consistent with the observed desorption of material upon annealing **[Figure 10h].**

The evolution of L^v with [(PRSH)PbI_3] is presented, for (200) α -, (210) α -, (020) α -, and (110) β-oriented crystallites, in **Figure S12.** In this case, the evolution of L^v is not the same for all reflections and also the effect of annealing is not the same: sometimes it induces an increase in L^v and sometimes a reduction. L^v of the β phase increases with %DMSO and the annealing induces a slight reduction but not for 80 % DMSO, where it increases to ~90 nm.

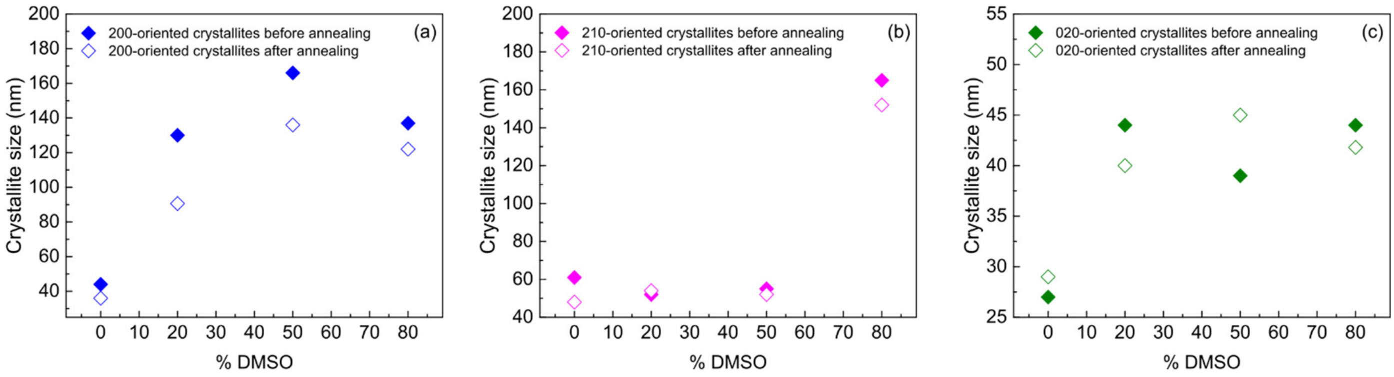

Figure S11. Bottom bound for the mean crystallite size of 200- (a), 210- (b), and 020-oriented crystallites (c) in (PRSH)$PbBr_3$ films before and after annealing, as a function of %DMSO in DMF.

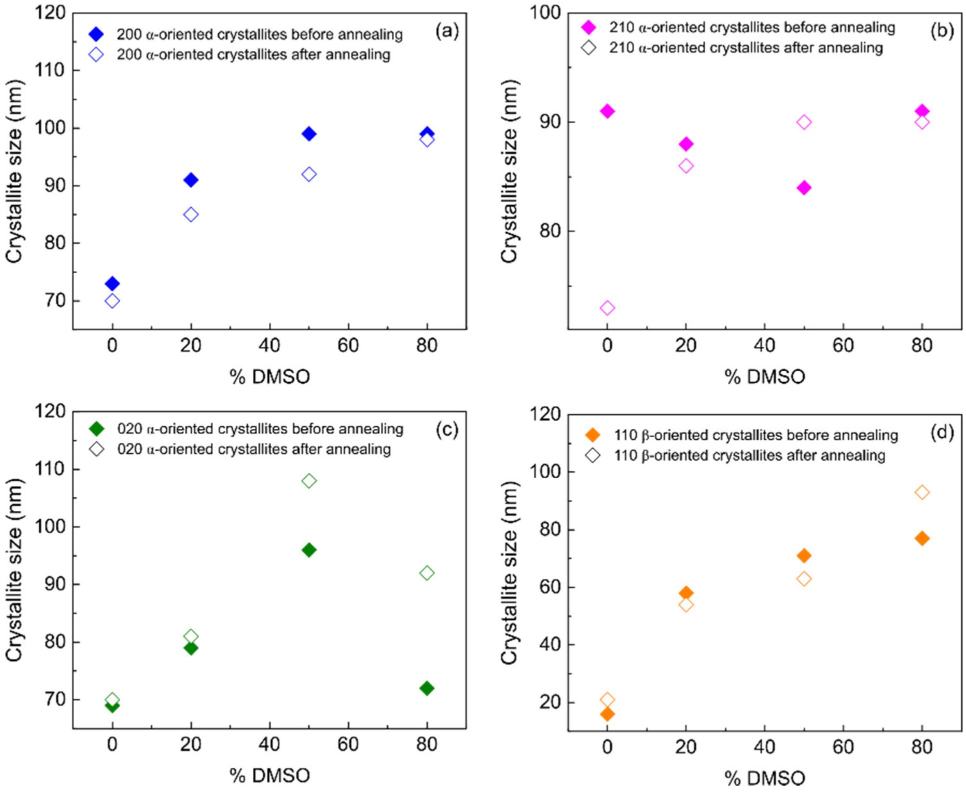

Figure S12. Bottom bound for the mean crystallite size of 200 α - (a), 210 α - (b), 020 α - (c) and 110 β -oriented crystallites (d) in $(PRSH)PbI_3$ films before and after annealing, as a function of %DMSO in DMF.

8. *In-situ* 2D-GIXD mapes recorded during thermal annealing

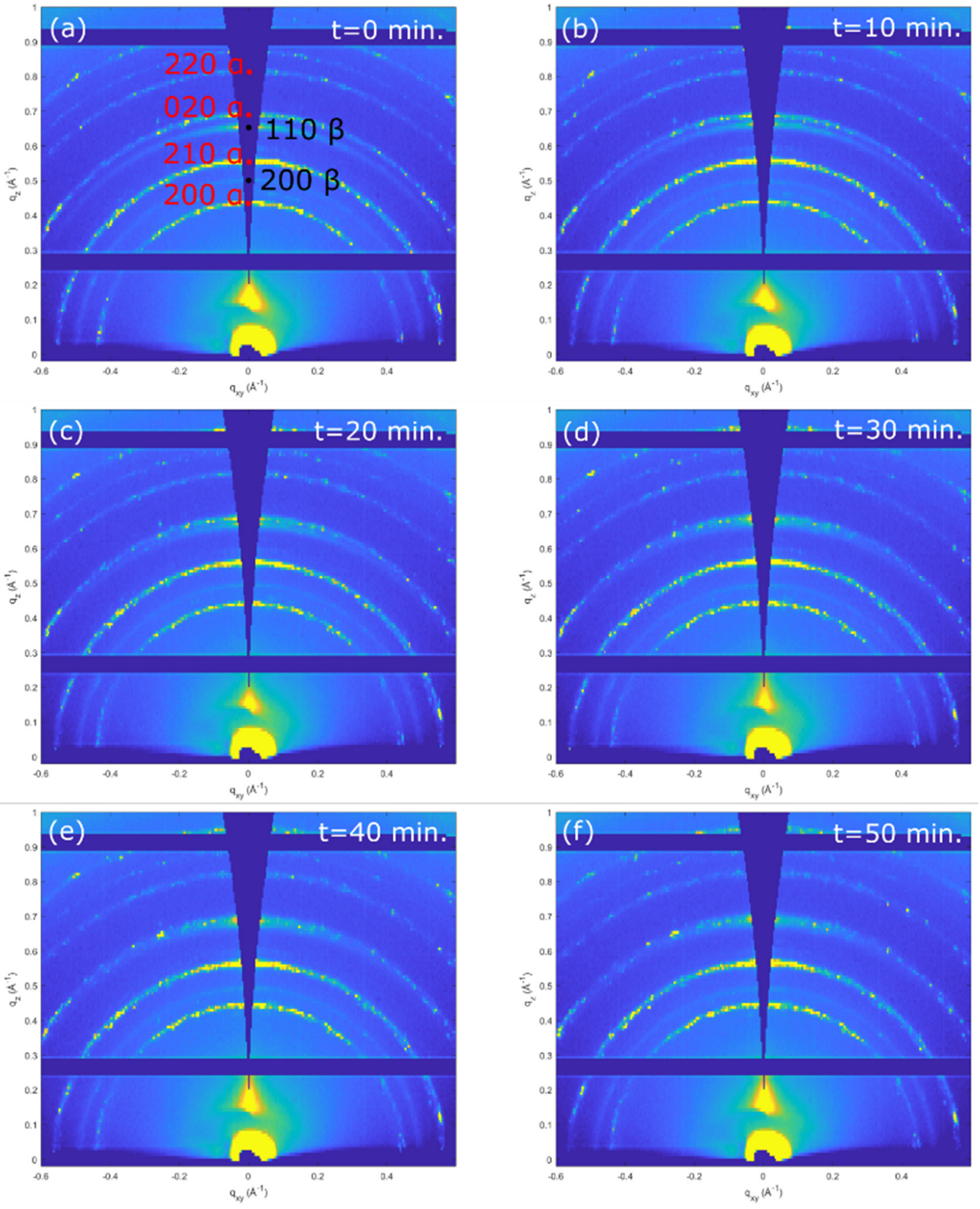

Figure S13. 2D-GIXD images of (PRSH)PbI_3 film prepared with DMF taken in-situ after 0 (a), 10 (b), 20 (c), 30 (d), 40 (e), and 50 minutes (f) while heating the sample to the constant temperature of 130 **°C**. t = 0 minutes corresponds to the time at which the sample reaches the target temperature of 130 °C.

Video: in-situ GIXD_(PRSH)PbI_3: Corresponding video reporting the series of the 2D-GIXD images recorded during in-situ heating at 130°C. The images are recorded every 90 sec.

9. Specular XRD patterns of (PRSH)PbX3 films before and after aging

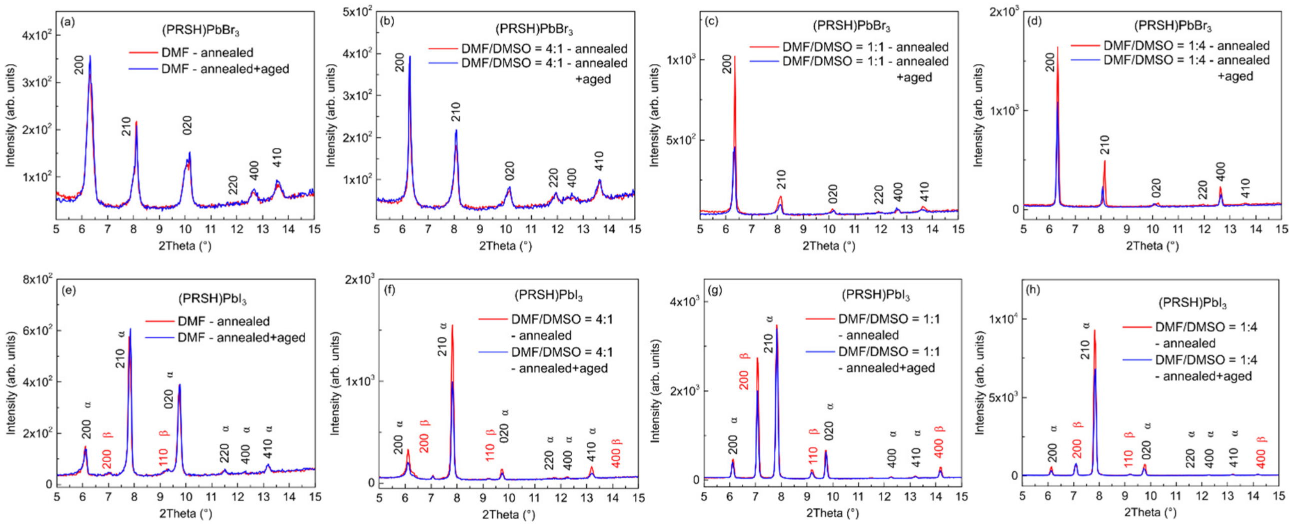

Figure S14. Specular XRD patterns of the annealed (PRSH)PbBr3 (a-d) and (PRSH)PbI3 (e-h) films taken before and after aging (red and blue traces, respectively), as a function of the used solvent.

10. Specular XRD patterns of (PRSH)PbX3 films under exposition to UV light

A series of 30 XRD profiles has been collected during UV light exposition, lasting 30 minutes each, for a total exposition time of 15 hours. Since no change is observed in the recorded diffractograms upon sample illumination, here we report the first and last recorded profiles, i.e. before UV irradiation and after 15 hours exposition.

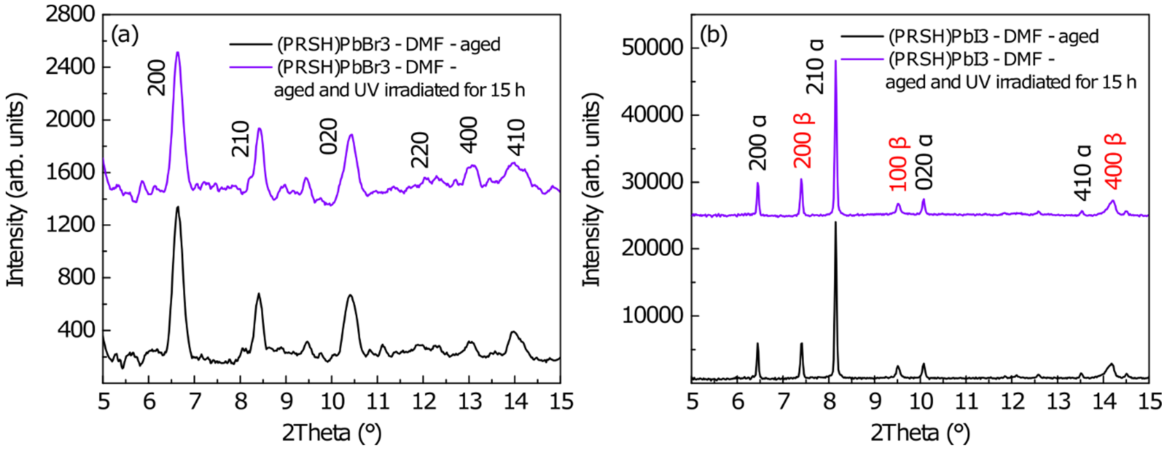

Figure S15. Specular XRD patterns for the aged DMF-only (PRSH)PbBr3 (a) and (PRSH)PbI3 (b) films taken before (black lines) and after exposition to UV light for 15 h (λ = 380 nm, power density = 600 mW/cm^2) (purple lines).

11. Specular XRD patterns of (PRSH)PbX3 films under different relative humidity conditions

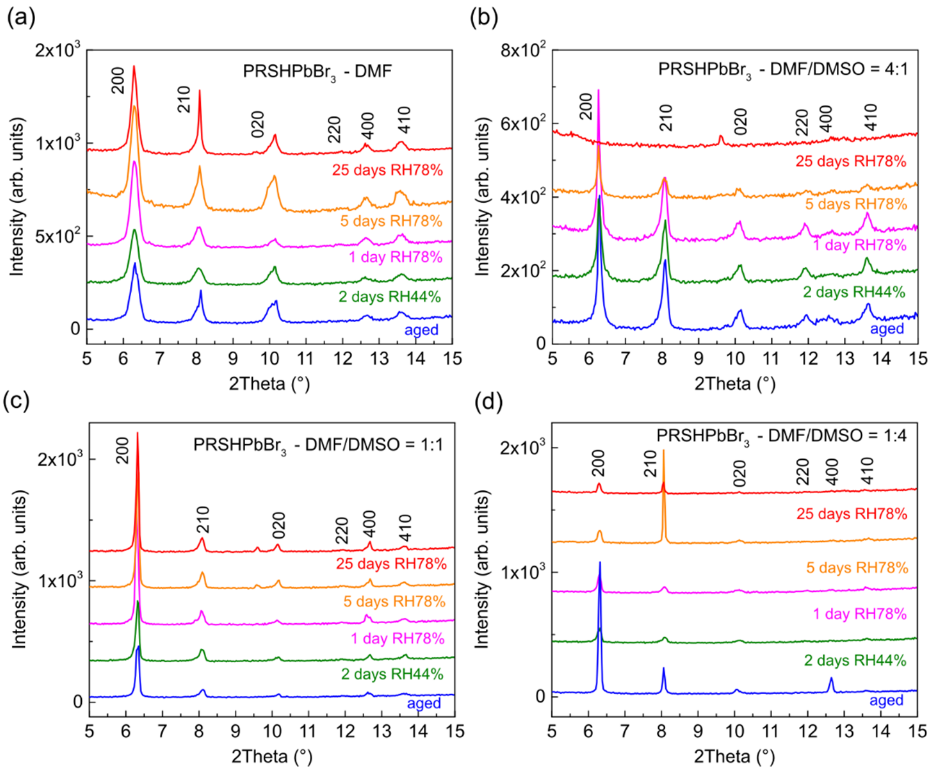

Figure S16. Specular XRD patterns taken on the aged (PRSH)PbBr3 films after sequential exposition to 44% relative humidity for 2 days, and to 78% relative humidity for 1, 5, and 25 days.

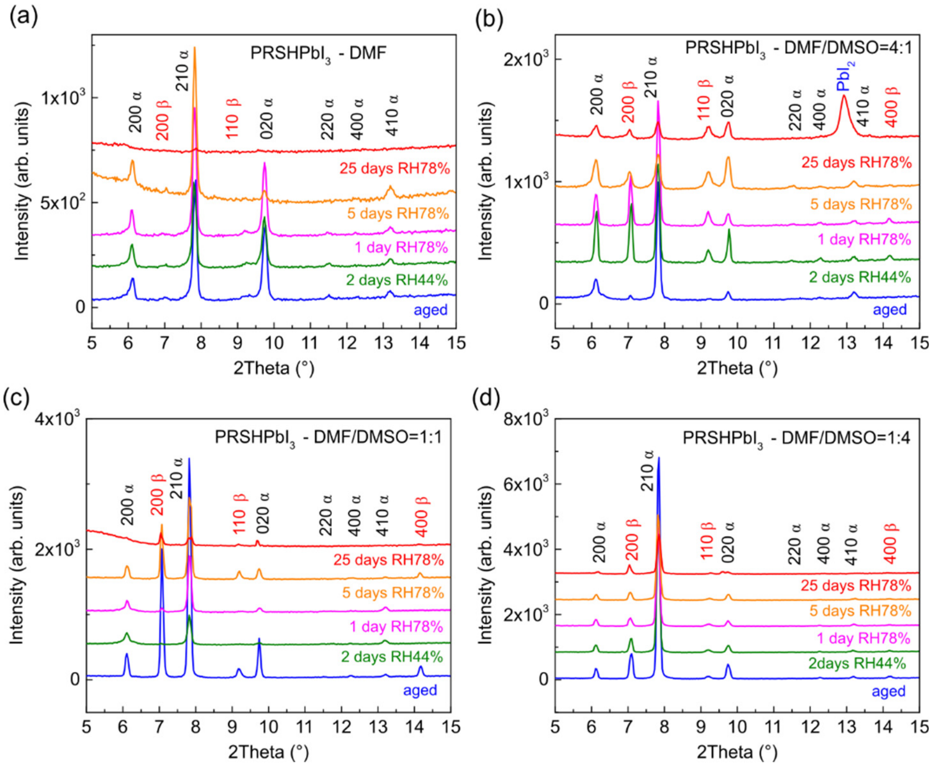

Figure S17. Specular XRD patterns taken on the aged (PRSH)PbI3 films after sequential exposition to 44% relative humidity for 2 days, and to 78% relative humidity for 1, 5, and 25 days.

SI References:

1. C. F. Macrae, I. Sovago, S. J. Cottrell, P. T. A. Galek, P. McCabe, E. Pidcock, M. Platings, G. P. Shields, J. S. Stevens, M. Towler and P. A. Wood, *Mercury 4.0: from visualization to analysis, design and prediction*, J. Appl. Cryst., 53, 226-235, 2020
